# Supplementary material for: Retroviral Replicating Vectors Mediated Prodrug Activator Gene Therapy in a Gastric Cancer Model
Source: Int J Mol Sci. 2023 Oct 2;24(19):14823. doi: 10.3390/ijms241914823 (PMC10573151; doi:10.3390/ijms241914823)
Supplement: Supplementary file 1 [file ijms-24-14823-s001.zip › ijms-2569236-supplementary.pdf]

Table S1: Human gastric cancer cell lines used in this study

name	ID number	Type	Tissue site of collection
HGC-27	RCB0500	Undifferentiated carcinoma	Metastatic lymph node
KATO-III	RCB2088	Signet ring carcinoma	Stomach
MKN-7	RCB3687	Highly differentiated tubular adenocarcinoma	Stomach
MKN-45	RCB1001	Poorly differentiated gastric adenocarcinoma	Stomach, Liver metastasis
MKN-74	RCB1002	Moderately differentiated adenocarcinoma	Stomach, Liver metastasis

Table S2: Abbreviation list

5-FC	5-fluorocytosine
5-FU	5-fluorourasil
AMLV	amphotropic murine leukemia virus
CD	yeast cytosine deaminase
DMEM	Dulbecco’s modified Eagle’s medium
FACS	fluorescence-activated cell sorter
FBS	fetal bovine serum
GALV	gibbon ape leukemia virus
GAPDH	glyceraldehyde-3-phosphate dehydrogenase
GC	gastric cancer
GFP	Green Fluorescent Protein
HSV-tk	herpes simplex virus thymidine kinase
IRES	internal ribosome entry site
MOI	multiplicity of infection
PBS	phosphate-buffered saline
PiT	inorganic phosphate transporter
qPCR	quantitative polymerase chain reaction
RPMI 1640	Roswell Park Memorial Institute 1640 medium
RRV	retroviral replicating vectors
RT-PCR	real-time polymerase chain reaction
SD	standard deviation
SE	standard error
TU	transduction units
